# Complement C1q Activates Tumor Suppressor WWOX to Induce Apoptosis in Prostate Cancer Cells

**DOI:** 10.1371/journal.pone.0005755

**Published:** 2009-06-01

**Authors:** Qunying Hong, Chun-I Sze, Sing-Ru Lin, Ming-Hui Lee, Ruei-Yu He, Lori Schultz, Jean-Yun Chang, Shean-Jen Chen, Robert J. Boackle, Li-Jin Hsu, Nan-Shan Chang

**Affiliations:** 1 Guthrie Research Institute, Laboratory of Molecular Immunology, Sayre, Pennsylvania, United States of America; 2 Department of Pathology, National Cheng Kung University Medical College, Tainan, Taiwan, Republic of China; 3 Department of Anatomy and Cell Biology, National Cheng Kung University Medical College, Tainan, Taiwan, Republic of China; 4 Institute of Molecular Medicine, National Cheng Kung University Medical College, Tainan, Taiwan, Republic of China; 5 Department of Engineering Science, National Cheng Kung University, Tainan, Taiwan, Republic of China; 6 Section of Oral Biology, Department of Stomatology, Medical University of South Carolina, Charleston, South Carolina, United States of America; 7 Department of Microbiology and Immunology, National Cheng Kung University Medical College, Tainan, Taiwan, Republic of China; 8 Center for Gene Regulation and Signal Transduction Research, National Cheng Kung University Medical College, Tainan, Taiwan, Republic of China; 9 Department of Neuroscience and Physiology, SUNY Upstate Medical University, Syracuse, New York, United States of America; Dresden University of Technology, Germany

## Abstract

**Background:**

Tissue exudates contain low levels of serum complement proteins, and their regulatory effects on prostate cancer progression are largely unknown. We examined specific serum complement components in coordinating the activation of tumor suppressors p53 and WWOX (also named FOR or WOX1) and kinases ERK, JNK1 and STAT3 in human prostate DU145 cells.

**Methodology/Principal Findings:**

DU145 cells were cultured overnight in 1% normal human serum, or in human serum depleted of an indicated complement protein. Under complement C1q- or C6-free conditions, WOX1 and ERK were mainly present in the cytoplasm without phosphorylation, whereas phosphorylated JNK1 was greatly accumulated in the nuclei. Exogenous C1q rapidly restored the WOX1 activation (with Tyr33 phosphorylation) in less than 2 hr. Without serum complement C9, p53 became activated, and hyaluronan (HA) reversed the effect. Under C6-free conditions, HA induced activation of STAT3, an enhancer of metastasis. Notably, exogenous C1q significantly induced apoptosis of WOX1-overexpressing DU145 cells, but not vehicle-expressing cells. A dominant negative and Y33R mutant of WOX1 blocked the apoptotic effect. C1q did not enhance p53-mediated apoptosis. By total internal reflection fluorescence (TIRF) microscopy, it was determined that C1q destabilized adherence of WOX1-expressing DU145 cells by partial detaching and inducing formation of clustered microvilli for focal adhesion particularly in between cells. These cells then underwent shrinkage, membrane blebbing and death. Remarkably, as determined by immunostaining, benign prostatic hyperplasia and prostate cancer were shown to have a significantly reduced expression of tissue C1q, compared to age-matched normal prostate tissues.

**Conclusions/Significance:**

We conclude that complement C1q may induce apoptosis of prostate cancer cells by activating WOX1 and destabilizing cell adhesion. Downregulation of C1q enhances prostate hyperplasia and cancerous formation due to failure of WOX1 activation.

## Introduction

Hyaluronic acid or hyaluronan (HA) participates in a remarkably multitude of cellular and physiologic events, including embryonic development, morphogenesis, differentiation, inflammation, wounding healing, immune response, and cancer progression and metastasis [1]–[4]. HA is involved in the initiation and progression of inflammation at both cellular and extracellular levels [5], [6]. At the cellular levels of inflammation, HA recruits neutrophils, activates macrophages and stimulates dendritic cell maturation. However, in serum HA may interact with complement proteins. Naturally occurring polysulfated glycosaminoglycans, such as heparin, restrict serum complement activation via both classical and alternative pathways during inflammation [7]–[10]. The potency of glycosaminoglycans in inhibiting complement activation depends upon their extent and positions of polysulfation. Unless conformationally altered, non-sulfated HA, even at high concentrations (1–5 mg/ml), cannot restrict complement activation [7], [11]–[13]. Induction of serum complement activation has been considered as important strategies in killing cancer cells *in vivo* [14], [15]. Cancer cell-derived inhibitors for blocking early complement components are known to enhance cancer growth [16]. Expression of the alternative pathway inhibitor factor H in lung cancer cells appears to be critical for their survival [17]. Nonetheless, the functional role of each individual serum complement component in regulating cancer cell survival is largely unknown.

Like other tissues, prostate is exposed to exudates from the blood, which contains low levels of circulating complement proteins. Whether complement proteins control prostate cell growth and hyperplasia with age is unknown. Here, by utilizing sera with selected deletion of complement proteins, we investigated whether each individual complement protein regulates the activation of tumor suppressors and kinase proteins in human prostate DU145 cells. These proteins include extracellular-signal regulated kinase/mitogen-activated protein kinase (ERK or MAPK) [18], WW domain-containing oxidoreductase (WWOX, FOR, or WOX1) [19]–[21], p53 [22], c-Jun *N*-terminal kinase (JNK1), and signal transducer and activator of transcription 3 (STAT3) [23]–[25]. We determined that without serum complement C1q or C6, the basal levels of activation or nuclear accumulation of ERK and WOX1 were reduced significantly, whereas constitutive activation of JNK1 occurred. WOX1 is a tumor suppressor and proapoptotic protein [19]–[21; reviews]. Interestingly, in the absence of serum complement C9, constitutive p53 activation occurred. High molecular size HA significantly induced the activation of STAT3 in DU145, only when C1q was absent in serum. STAT3 promotes prostate cancer invasion [23]–[25]. Finally, C1q alone was able to activate ectopic WOX1 in killing prostate cancer cells. We discussed the likely scenario for circulating or pericellular HA and complement proteins in regulating cancer cell growth and death via activation of p53, WOX1, JNK1, and STAT3.

## Results

### Complement C1q activates tumor suppressor WWOX/WOX1 in human prostate DU145 cells

We determined whether C1q activates endogenous WOX1 for nuclear accumulation. Substantial evidence reveals that WOX1 is a tumor suppressor [19]–[21], [26]. Phosphorylation of WOX1 at Tyr33 (p-WOX1) is essential for its apoptotic activity both *in vitro* and *in vivo* [27]–[31]. Nonetheless, during the initial hyperplasia and cancerous stages, there is a significant upregulation of WOX1 expression and Tyr33 phosphorylation in prostate, skin and breast, and that the expression is reduced dramatically during malignancy and metastasis *in vivo* [21], [29], [32]. Murine WOX1/Wwox is critical for postnatal survival, insofar as the knockout mice could survive for only one month [20], [33]. Also, this protein is essential for normal bone metabolism [33]. The mechanisms regarding the control for WOX1 to exert prosurvival or proapoptotic functions remain to be established.

**Figure 1:**
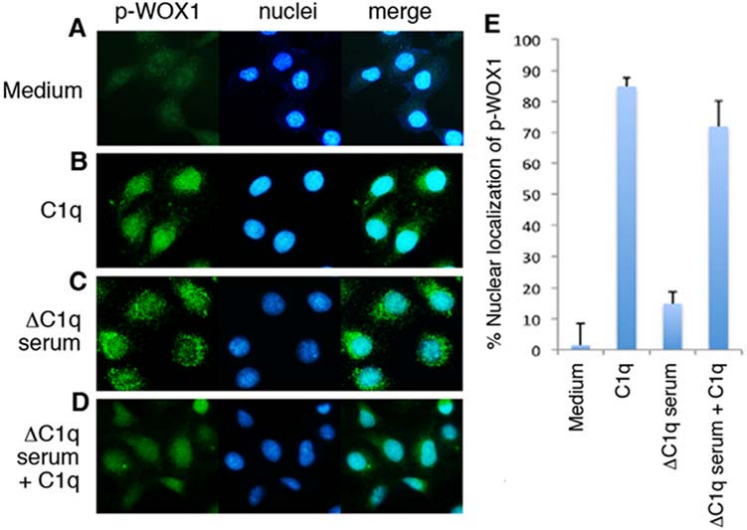
Exogenous complement C1q increases p-WOX1 nuclear accumulation in human prostate DU145 cells. (A,B) DU145 cells were grown on cover glass and cultured overnight in 10% heat-inactivated fetal bovine serum. The cells were then starved under serum-free conditions for 1 hr, followed by treating with or without purified C1q (1 µg/ml) for 1 hr. Localization of endogenous Tyr33-phosphorylated WOX1 (p-WOX1) was determined by immunofluorescence microscopy. (C,D) In addition, the starved cells were then cultured in 1% C1q-depleted human serum (ΔC1q serum) for 1 hr, in the presence or absence of exogenous C1q (1 µg/ml). (E) Presence of p-WOX1 in the nuclei is shown from counting ∼100 cells in 3 experiments (mean±standard deviation).

Human DU145 cells were cultured overnight in the presence of heat-inactivated fetal bovine serum (10%), followed by starvation for 1 hr without serum. These cells were then treated with purified C1q for 1 hr. Localization of p-WOX1 was determined by immunofluorescence microscopy. These starved cells had very low levels of cytoplasmic p-WOX1 (Fig. 1A). Exogenous C1q rapidly induced accumulation of p-WOX1 in the nuclei (Fig. 1B). In comparison, when the starved cells were cultured in 1% C1q-depleted (ΔC1q) human serum for 1 hr, p-WOX1 was mainly localized in the cytoplasm (Fig. 1C). Reconstitution of ΔC1q serum with purified C1q rapidly induced p-WOX1 accumulation in the nuclei (Fig. 1D).

### Complement C1q activates ectopic WOX1 for inducing apoptosis of DU145 cells

**Figure 2:**
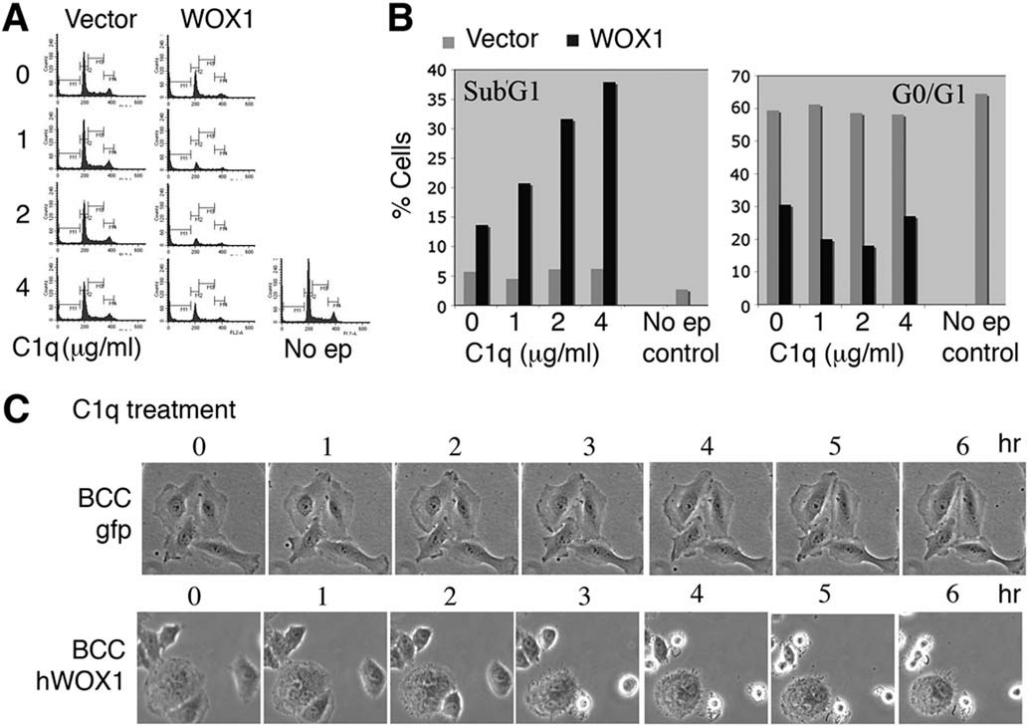
Complement C1q increases ectopic WOX1-induced apoptosis of DU145 cells. (A) DU145 cells were transfected with EGFP-WOX1 (tagged with EGFP) or EGFP alone by electroporation, cultured overnight, and then treated with purified C1q (1 µg/ml) for 24 hr. C1q enhanced WOX1-induced apoptosis of DU145 cells (see increases in SubG1 phase). In vector controls, C1q did not enhance apoptosis in DU145 cells overexpressing EGFP. (B) A representative data set from 3 experiments is shown in the bar graph. Similar results were observed by transfecting DU145 cells with various amounts of WOX1, followed by treating with 1 µg/ml C1q overnight (see Supplementary Fig. S1). WOX1: EGFP-WOX1. Vector: EGFP only. No ep: cells without electroporation. (C) Stable transfectants of BCC cells for expressing EGFP or EGFP-hWOX1 (human WOX1/WWOX) were established. By time-lapse microscopy, C1q (1 µg/ml) induced death of hWOX1-expressing cells, but not EGFP-expressing cells. These hWOX1-expressing cells appeared to undergo typical apoptosis, as they exhibited cell shrinkage and membrane blebbing in a time-related manner. A representative data is shown from 5 experiments.

We determined whether C1q activates ectopic WOX1 for inducing apoptosis. DU145 cells were transfected with EGFP-WOX1 (tagged with EGFP) or EGFP alone by electroporation. These cells were cultured overnight (in 10% heat-inactivated fetal bovine serum), followed by treating with purified C1q for 24 hr. C1q enhanced WOX1-induced apoptosis and growth suppression of DU145 cells in a dose-related manner, as revealed by an increased cell population at the SubG1 phase and a reduced population at the G0/G1 phase of the cell cycle (Figs. 2A and 2B and Supplementary Fig. S1). In controls, C1q did not induce apoptosis in DU145 cells overexpressing EGFP vector only (Figs. 2A and 2B).

The enhancement of apoptosis by C1q was not due to its activation of complement cascade in the fetal bovine serum, insofar as the serum was heat-inactivated. Also, under serum-free conditions, exogenous C1q enhanced ectopic WOX1-mediated apoptosis (data not shown). These observations suggest that WOX1 is a downstream effector of C1q-mediated apoptosis, without involvement of complement activation.

Under similar experimental conditions, we showed that C1q increased apoptosis in cells overexpressing WOX1. These included breast MCF7 (Supplementary Fig. S2), and neuroblastoma SH-SY5Y (Supplementary Fig. S3) and SK-N-SH cells (data not shown). Again, no effect was observed using cells overexpressing EGFP only.

In parallel, stable transfectants of skin basal cell carcinoma (BCC) cells for expressing EGFP or human WWOX/WOX1 (hWOX1 tagged with EGFP) were established using G418 selection. By time-lapse microscopy, we showed that BCC cells expressing EGFP were resistant to C1q-mediated cell death, whereas hWOX1-expressing cells were sensitive to C1q (Fig. 2C). These hWOX1-expressing cells appeared to exhibit typical morphology of apoptosis, including cell shrinkage and membrane blebbing.

### *N*-terminal Tyr33-phosphorylated WW domain of WOX1 is responsible for C1q-induced apoptosis of DU145 cells

**Figure 3:**
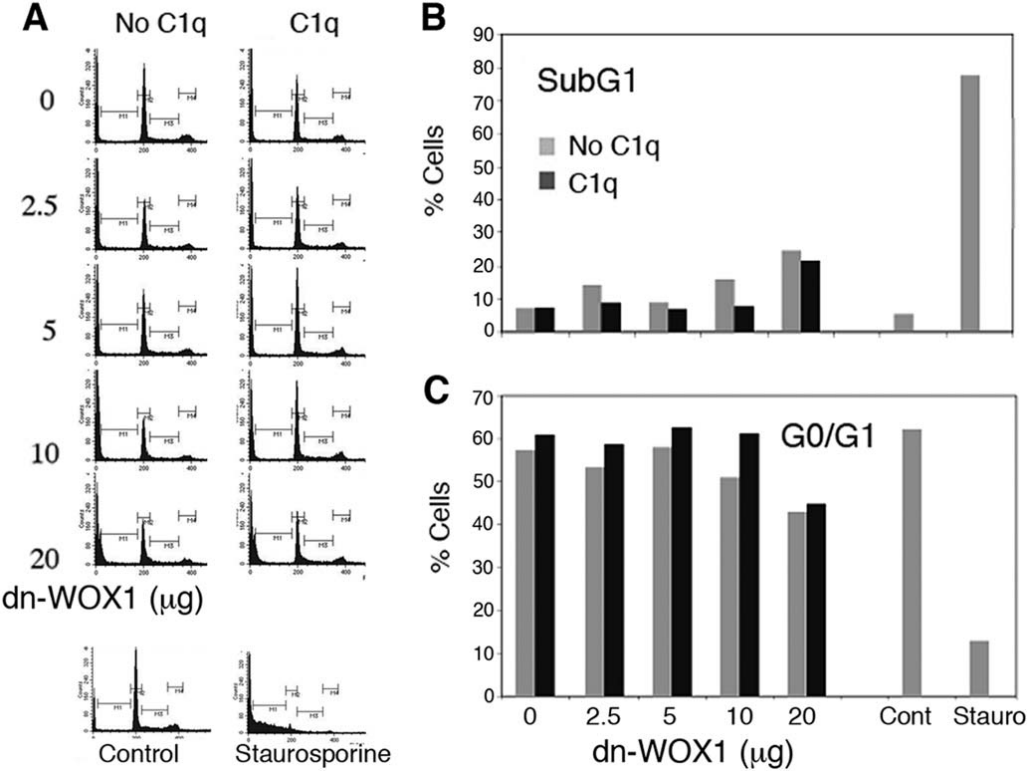
Dominant-negative WOX1 (dn-WOX1) blocks C1q-mediated apoptosis of DU145 cells. (A) DU145 cells were electroporated with various amount of dn-WOX1 (EGFP tag) or EGFP alone, followed by culturing for 24 hr. dn-WOX1-expressing cells were treated with C1q (1 µg/ml) overnight, and cell cycle analysis was performed. EGFP-expressing cells were treated similarly (data not shown). In addition, non-transfected control cells were treated with or without staurosporine (1 µM) overnight. (B,C) A representative data set for both SubG1 and G0/G1 phases is shown (from 3 experiments).

We determined which domain(s) in WOX1 participates in C1q-mediated apoptosis of DU145 cells. Human and murine WWOX/FOR/WOX1 is composed of two *N*-terminal WW domains, a nuclear localization sequence, and a *C*-terminal short-chain alcohol dehydrogenase/reductase (SDR) domain [19–21,34; reviews]. A dominant negative-WOX1 (dn-WOX1) was designed previously, with alterations in the *N*-terminal first WW domain [27]. dn-WOX1 is known to block the apoptotic function of p53 and prevent phosphorylation of endogenous WOX1 at Tyr33 [27]. When DU145 cells were transiently overexpressed with dn-WOX1 (EGFP tag), the cells resisted C1q-induced apoptosis (Fig. 3). In controls, cells were transfected with an EGFP vector only, and the cells did not undergo apoptosis in response to C1q (data not shown or see Fig. 2). In positive controls, non-transfected cells were treated with staurosporine to induce apoptosis (Fig. 3).

**Figure 4:**
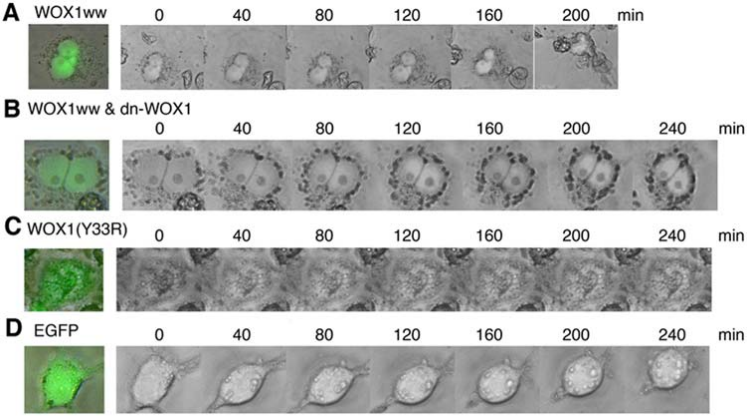
The *N*-terminal WW domain of WOX1 is associated with C1q-induced apoptosis of DU145 cells. (A) Live DU145 cells-expressing the *N*-terminal WW domain (WOX1ww; EGFP tag) were treated with C1q (1 µg/ml), followed by recording morphological changes by automatic time-lapse microscopy (one frame per 10 min). Cell shrinkage and nuclear condensation occurred approximately 100–130 min upon exposure of cells to C1q. (B) When cells were cotransfected with WOX1ww and dn-WOX1, C1q-induced apoptosis was significantly reduced. (C) Alteration of Tyr33 to Arg33 in WOX1 did not cause C1q-mediated cell death. (D) No apoptosis was observed in cells expressing EGFP only upon exposure to C1q. Compared to the above experiments, C1q concentration was increased for the time-lapse microscopy experiments. A representative data set is shown from 5 experiments. Approximately 100 cells were examined at the end of time-lapse microscopy. A merged photo of the cell expressing green fluorescence and the bright field image is shown (prior to challenge with C1q).

Thus, based on the above observations, the *N*-terminal WW domain of WOX1 is likely to be responsible for C1q-induced activation of WOX1 for killing cancer cells. DU145 cells were transfected with the *N*-terminal WW domain of WOX1 (WOX1ww with an EGFP tag) or EGFP only by electroporation and cultured for 24 hr. By time-lapse microscopy of live cells, exogenous C1q induced apoptosis of cells expressing the WW domains (Fig. 4A). Cell shrinkage and nuclear condensation occurred approximately 100–130 min upon exposure of cells to C1q. This is a typical event of apoptosis. When DU145 cells were cotransfected with WOX1ww and dn-WOX1, C1q-induced apoptosis was lessened (Fig. 4B). Tyr33 phosphorylation in WOX1 plays a key role in apoptosis both *in vitro* and *in vivo* [27]–[31]. We altered Tyr33 to Arg33 in the first WW domain [27], [35], and determined that C1q did not mediate apoptosis when cells expressed this mutant protein (Fig. 4C). In the vector control cells, no apoptosis was observed (Fig. 4D). C1q-mediated cell death was not observed in these control cells after a longer incubation for more than 8–24 hr, which is similar to the aforementioned observations (Fig. 2).

**Figure 5:**
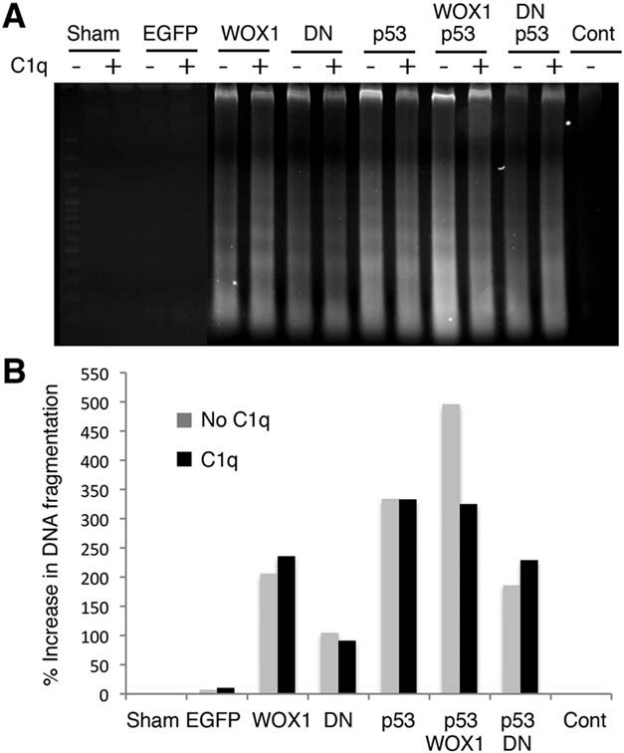
C1q-mediated internucleosomal DNA fragmentation in WOX1 and/or p53-expressing DU145 cells. (A) DU145 cells were electroporated with cDNA expression constructs of WOX1, dn-WOX1 (DN), and/or p53. 24 hr later, the cells were exposed to C1q for 8 hr. In appropriate controls, cells were electroporated with medium (Sham) or without electroporation (Cont). No DNA fragmentation was shown in these controls. C1q increased the DNA fragmentation in cells expressing WOX1, but not p53. C1q suppressed p53/WOX1-increased DNA fragmentation. dn-WOX1 inhibited cell death caused by p53. (B) The intensity of DNA fragmentation was quantified by Photoshop, and averaged results shown in the bar graph were from two experiments. The “Sham” control (without C1q treatment) is regarded as 0%.

The C1q/WOX1-induced apoptosis was further confirmed by internucleosomal DNA fragmentation analysis using agarose gel electrophoresis. The results showed the cleaved DNA ladders as induced by C1q in WOX1-expressing DU145 and SH-SY5Y cells (Fig. 5 and Supplementary Fig. S4). In appropriate control cells expressing EGFP or nothing, C1q did not induce DNA fragmentation (Fig. 5 and Supplementary Fig. S4).

### C1q does not increase p53-mediated apoptosis

We have shown that tumor suppressor p53 physically interacts with WOX1, and both proteins may cause apoptosis in a synergistic manner [26], [27], [30]. Importantly, WOX1 stabilizes p53 and prevents its degradation [30]. We determined the role of p53 and WOX1 in C1q-regulated cell death. When DU145 cells were transiently overexpressed with p53, C1q did not significantly increase the extent of DNA fragmentation (Figs. 5A and 5B). In combination, both p53 and WOX1 increased the DNA fragmentation. Intriguingly, C1q suppressed the DNA fragmentation in the p53/WOX1-expressing cells. In agreement with our previous observations [27], dn-WOX1 was shown to inhibit p53-mediated DNA fragmentation (Figs. 5A and 5B).

### Ectopic WOX1 induces formation of clustered microvilli in between DU145 cells and C1q enhances the cluster formation

**Figure 6:**
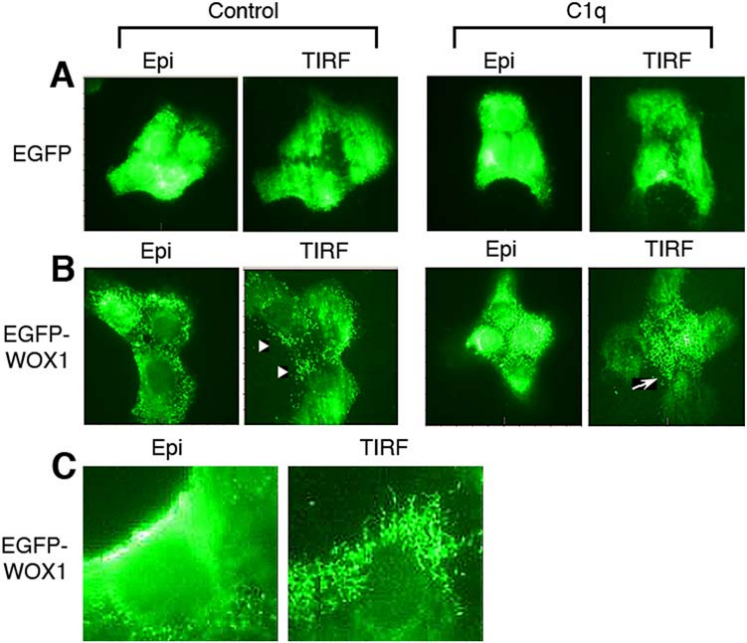
C1q induces cluster formation of WOX1-containing microvilli on the surface in between DU145 cells. (A) When DU145 cells were transfected with EGFP and cultured overnight, these cells adhered flatly on the cover glass surface, as determined by TIRF microscopy for measuring the green fluorescence on the cell membrane and cytoskeletal areas (600× magnification). Epi: epifluorescence. (B) In contrast, transiently overexpressed EGFP-WOX1 induced the formation of punctates, as appeared in clusters, on the cell surface (see arrowheads; 600× magnification). C1q significantly increased the punctate formation, particularly in between cells (see arrow). Increases in the numbers of punctuates are approximately 3–6 fold. (C) The clustered punctates, which are rich in EGFP-WOX1 expression, are indeed microvilli on the cell surface (600× magnification by microscopy and 3x digital magnification).

We examined the cell morphological alterations caused by ectopic expression with EGFP or EGFP-WOX1 in DU145 cells, plus the effect of C1q. Time-lapse microscopy showed that C1q induced shrinkage and membrane blebbing of WOX1-expressing DU145 and BCC cells during treatment for 2–4 hr or longer (Figs. 2C and 4A). When DU145 cells were overexpressed with EGFP and cultured overnight, these resting cells adhered flatly on the cover glass surface, as determined by total internal reflection fluorescence (TIRF) microscopy (Fig. 6A). TIRF imaging measures the protein dynamic events on the cell membrane and cytoskeletal areas [36], [37]. Interestingly, transiently overexpressed EGFP-WOX1 induced punctate formation on the cell surface, and these punctates, containing EGFP-WOX1, were mostly clustered in between cells (Fig. 6B; see arrowheads). At a higher magnification, these punctates were indeed microvilli, which are needed for focal adhesion (Fig. 6C). Notably, many of these WOX1-expressing cells adhered onto the cover glass surface mainly by pericellular microvilli, as their ventral areas were detached from the surface. During treatment for 1 hr, C1q significantly increased the formation of clustered microvilli in between cells (Fig. 6B; see arrow). This action appears to destabilize cell adhesion for subsequent shrinking, membrane blebbing and eventual death. In agreement with our recent report [38], WOX1 can be associated with cell surface hyaluronidase Hyal-2.

### C1q is expressed in the archival prostate tissue samples and is significantly downregulated in hyperplasia and cancerous prostate tissues

**Figure 7:**
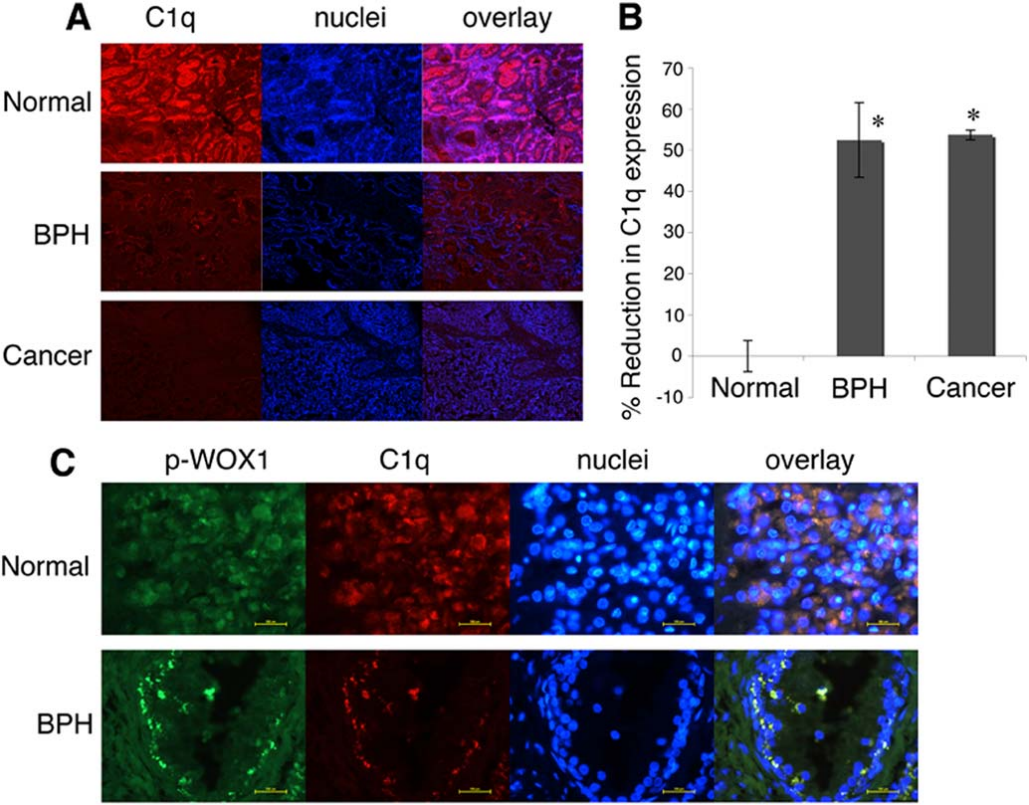
C1q is expressed in normal archival prostate tissue samples and is significantly downregulated in benign prostatic hyperplasia (BPH) and prostate cancer. (A) Prostate tissues, including normal prostate, BPH and prostate cancer, were stained with specific antibody against C1q and then with secondary fluorescent antibody. Nuclei were stained with DAPI (100× magnification). (B) C1q is significantly downregulated in BPH and prostate cancer, as compared to age-matched prostate tissues (*p*<0.0001, n = 5; Student's *t* test). When non-immune serum was used as the source for primary antibody, no signal was observed (data not shown). (C) C1q is expressed in the basal and epithelial cells of the archival normal prostate glands (400× magnification). Both C1q and p-WOX1 are colocalized in these cells. Nuclei were stained with DAPI. Similar results were observed with the archival gland cells from BPH. Compared to the normal prostate tissues, the exposure time for taking the pictures for BPH was increased. Scale bar, 100 µm.

The above observations suggest that serum- or tissue-derived complement C1q may induce WOX1 activation *in vivo*, thereby restricting cancerous progression. While alterations of human *WWOX* gene occur most frequently in prostate and breast [20], [21], [34], we examined the expression of C1q in archival prostate tissues from postmortem patients. By immunofluorescence microscopy, we determined that compared to age-matched prostate tissues, C1q is significantly downregulated in benign prostatic hyperplasia (BPH) and prostate cancer (Figs. 7A and 7B; 100× magnification). At a higher magnification, C1q was shown to express in the basal and epithelial cells of the archival prostate tissue samples, and C1q was colocalized with p-WOX1 in these cells (Fig. 7C).

### Serum complement C1q and C6 are essential for supporting basal phosphorylation of ERK and WOX1 in DU145 cells

**Figure 8:**
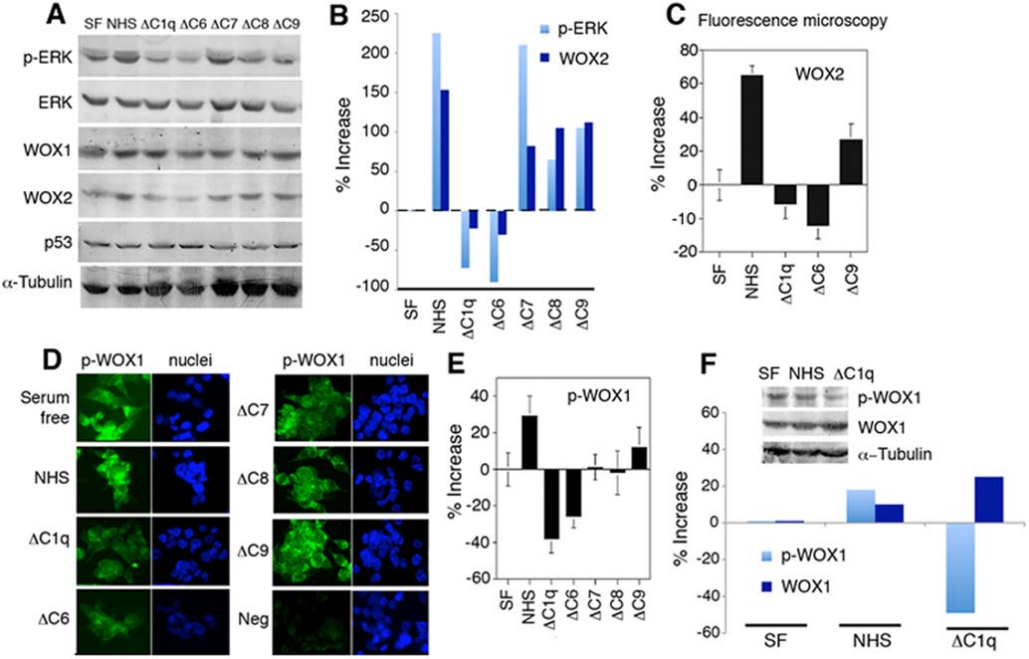
Complement C1q and C6 are essential for maintaining ERK and WOX1 activation and expression of isoform WOX2. (A,B) DU145 cells were cultured overnight under serum-free (SF) conditions, or in 1% normal human serum (NHS) or NHS depleted with C1q (ΔC1q), C6 (ΔC6), C7 (ΔC7), C8 (ΔC8), or C9 (ΔC9). Significant reduction of isoform WOX2 expression was observed in DU145 cells when cultured in ΔC1q or ΔC6 serum (versus SF controls; *p*<0.001; Student's *t* test), whereas ΔC7, ΔC8, or ΔC9 serum was less effective. Activation or phosphorylation of ERK (p-ERK) was dependent upon the presence of C1q or C6 in serum (versus SF controls; *p*<0.001; Student's *t* test). A representative set of data from 3 experiments is shown. The bar graphs show the average of 3 experiments. (C) The above cells were also grown on cover glass under identical serum conditions. By immunofluorescence microscopy, serum without C1q or C6 could not support the constitutive expression of WOX2 (*p*<0.0001, as versus SF or NHS; Student's *t* test). Approximately 200 cells were quantified individually under fluorescence microscopy, and the extent of fluorescence was subtracted (or normalized) from negative controls (cells stained with secondary antibody only). Nuclei were stained with DAPI. (D,E) The same cells, as indicated in (A), were stained with p-WOX1 antibody and the extent of protein expression was quantified. (F) Again, identical experiments were carried out for Western blotting. Under C1q-free conditions (ΔC1q serum), the basal activation of WOX1 was significantly reduced (∼50% reduction; *p*<0.001 from versus SF and NHS; Student's *t* test; n = 3).

We investigated whether downregulation of C1q *in vivo* may reduce the activation of tumor suppressors *in vitro*, thereby providing better survival for prostate cancer cells. DU145 cells were cultured overnight under serum-free conditions, in the presence of 1% normal human serum, or 1% human serum with depletion of C1q, C6, C7, C8, or C9. By Western blotting, we determined that both ΔC1q and ΔC6 sera could not support the constitutive expression of WOX2 (an isoform of WOX1) and p-ERK in DU145 cells (Figs. 8A and 8B), suggesting that serum C1q and C6 components are essential for the expression of these proteins. In comparison, component C7, C8 and C9 did not support the expression of WOX2 and p-ERK (Figs. 8A and 8B). Expression of ERK, WOX1, MEK1, and p53 was not significantly affected by the above-mentioned culture conditions (Figs. 8A and 8B; data not shown for MEK1).

To confirm the aforementioned observations, immunofluorescence microscopy was carried out. Cells from the above experiment were grown on cover glass under identical serum conditions, then fixed, and stained with specific fluorescence antibodies. Again, we determined that in the absence of serum C1q and C6, WOX2 expression was significantly downregulated in DU145 cells (Fig. 8C). Additionally, when DU145 cells were cultured in serum without C1q or C6, the levels of p-WOX1 were significantly decreased in DU145 cells, compared with other culture conditions, as determined by immunofluorescence microscopy (Figs. 8D and 8E). These observations were further confirmed by Western blotting, which revealed downregulation of p-WOX1 under C1q-free conditions (Fig. 8F). Under these conditions, p-WOX1 was present mainly in the cytoplasm of DU145 cells. No apoptosis was observed in these cells during 24 hr in culture, as determined by the morphology of nuclei stained with DAPI.

### Complement C9 depletion promotes p53 nuclear accumulation, and hyaluronan stimulates nuclear export

**Figure 9:**
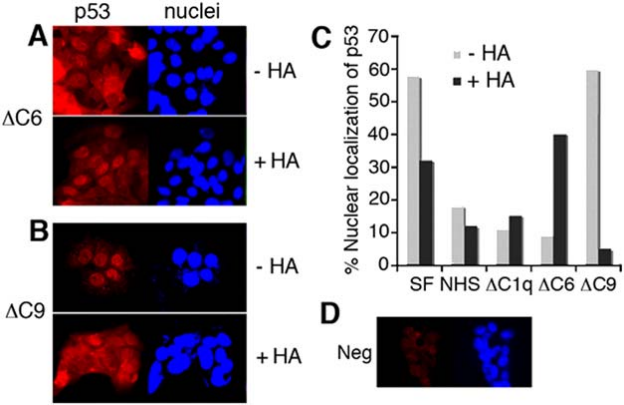
p53 accumulates in the nuclei of DU145 cells cultured in complement C9-free serum, and HA reverses the nuclear accumulation. DU145 cells were cultured overnight in each indicated serum with depletion of a specific complement protein. p53 localization in the cells was determined by immunofluorescence microscopy. (A) In the absence of complement C6 (ΔC6 serum), p53 was mainly present in the cytoplasm. High molecular size HA (50 µg/ml) induced nuclear accumulation of p53 during treatment for 1 hr. (B) In the absence of serum C9 (ΔC9 serum), p53 was mostly localized in the nuclei, and HA (50 µg/ml) induced nuclear export in 1 hr. (C) To determine nuclear localization of p53, approximately 200 cells were counted. Shown in the bar graph is an average of results form two experiments. (D) In negative controls, cells were stained with Texas Red-conjugated secondary antibody only. SF, serum free; NHS, normal human serum.

WOX1 physically interacts with p53 both *in vitro* and *in vivo*, and may induce apoptosis synergistically [21], [26], [27], [30]. Although exogenous C1q could not increase p53-mediated apoptosis (Fig. 5), we examined the effect of serum complement C1q and C6 in enhancing the basal levels of p53 nuclear accumulation or activation. When DU145 cells were grown in 1% normal serum or in serum without C1q or C6, accumulation of p53 in the nuclei was reduced, as compared to serum-free conditions (Fig. 9A and 9C). Remarkably, without serum C9, p53 was accumulated in the nuclei (Fig. 9B and 9C), suggesting that serum complement C9 is able to restrict p53 activation. Under C9-deficient conditions, exogenous high molecular weight HA induced nuclear export of p53 to the cytoplasm (Fig. 9B and 9C). In contrast, HA restored p53 nuclear accumulation under C6-free conditions (Fig. 9A and 9C).

### Serum complement C1q and C6 depletion induces constitutive JNK1 activation

**Figure 10:**
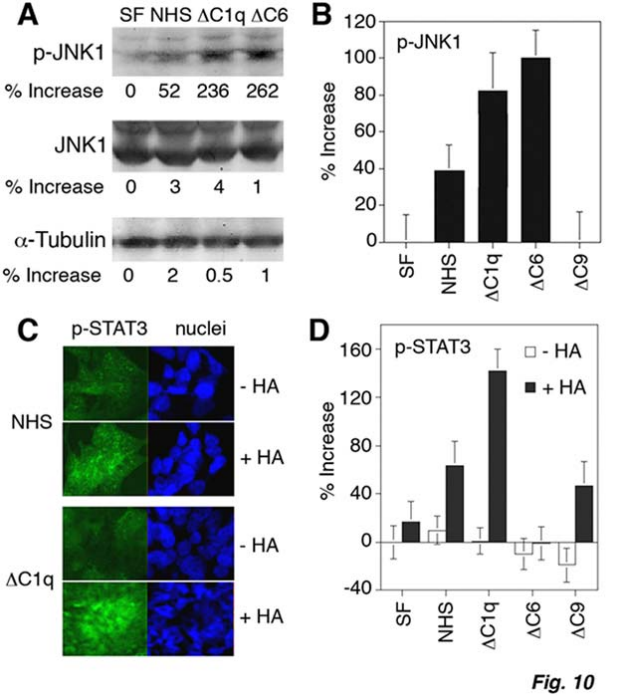
Serum C1q and C6 restrict constitutive JNK1 activation but have no effect on STAT3. (A) DU145 cells were cultured under serum-free (SF) conditions, or in 1% NHS, ΔC1q or ΔC6 serum for 16–24 hr. In the absence of C1q and C6, spontaneous JNK1 activation occurred (versus SF controls; *p*<0.001; Student's *t* test), as determined by Western blotting. A representative set of data is shown from 3 experiments. (B) Similar results were observed by immunofluorescence microscopy, which shows significant JNK1 activation or nuclear accumulation under serum C1q- and C6-free conditions (versus SF; *p*<0.001; Student's *t* test, n = 3). ΔC9 serum had no effect. Approximately 200 cells were counted from each experiment. (C,D) There was no STAT3 activation in DU145 cells under all the indicated serum conditions. Under C1q-free conditions, HA (50 µg/ml) effectively induced STAT3 phosphorylation in cells during treatment for 1 hr. Approximately 200 cells were counted from each experiment (n = 3).

We have shown that JNK1 physically interacts with WOX1, and that the binding is increased upon stimulation of cells with UV light or anisomycin (activator of JNK1) *in vitro* [27]. Interestingly, dopaminergic neurotoxin 1-methyl-4-phenyl-pyridinium (MPP^+^) reduces the binding of WOX1 with JNK1 in rat brains [31]. JNK1 blocks the apoptotic function of WOX1 *in vitro* [27], whereas the functional antagonism *in vivo* is unknown. JNK1 and isoforms are involved in stress and apoptotic responses, cell proliferation, and many types of diseases [39]. While the above results showed that complement C1q and C6 supported the activation of WOX1 (Fig. 8), these proteins are predicted to block JNK1 activation, and depletion of C1q and C6 from sera will cause JNK1 activation. Under similar experimental conditions, when DU145 cells were grown in ΔC1q or ΔC6 serum, constitutive JNK1 activation occurred, as determined by both Western blotting (Fig. 10A) and immunofluorescence microscopy (Fig. 10B).

### In the absence of C1q, HA induces STAT3 phosphorylation in DU145 cells

STAT3 activation plays a critical role in prostate cancer cell invasion [23]–[25]. HA also participates in cancer invasion, suggesting that HA activates STAT3 for promoting cancer metastasis. Similarly, DU145 cells were cultured overnight under various serum-free or -present conditions. These cells were then exposed to HA for 1 hr. No activation of STAT3 was observed using normal or complement-depleted sera (Fig. 10C and 10D). Notably, in the absence of C1q, HA induced STAT3 activation in DU145 cells (Fig. 10C and 10D), implying that HA may increase metastasis of C1q-deficient prostate cancer cells by upregulating STAT3 phosphorylation and suppressing activation of p53 and WOX1. All the above experiments were performed using medical grade HA from Lifecore. Similar results were observed using medical grade Healon (data not shown). These HA preparations are free of protein contamination.

## Discussion

The human serum complement cascade is traditionally considered as a powerful humoral immune system that protects against invading microorganisms. Nonetheless, the functional properties of each individual complement component are largely unknown. In this study, we have demonstrated for the first time that C1q is expressed in the prostate. Remarkably, C1q expression is significantly reduced in benign prostatic hyperplasia and prostate cancer. This event is critical for prostate cancer to grow and develop. Our supporting evidence is that complement C1q and C6 sustain the activation of tumor suppressor WOX1, which is needed for blocking cancer cell proliferation. WOX1 colocalizes with C1q in the basal and epithelial cells of prostate tissues. Functional significance of this regard remains to be established.

In cell model experiments, we determined that C1q activates ectopic WOX1 in killing DU145 cells. The sequential event is as follows: 1) Cells overexpressing WOX1 attach evenly onto the surface of cover glass (determined by TIRF microscopy), whereas approximately 50% of cells may “tiptoe” on the glass surface via formation of microvilli for focal adhesion; 2) C1q dramatically increases the formation of microvilli as clusters in between cells. WOX1 can be found in both the focal adhesion areas and in the nuclei; 3) Cells then undergo shrinkage, membrane blebbing, nuclear condensation and DNA fragmentation. Exposure of phosphatidylserine (PS) on the cell surface is barely detectable, raising the question whether PS exposure is essential for C1q/WOX1-induced cell death. WWOX/WOX1 has been shown to localize in the cell membrane/cytoskeleton area [38], [40]. We determined that dominant negative and Y33R mutant of WOX1 blocked the cell death, indicating that Tyr33 activation is essential for C1q/WOX1-mediated apoptosis. This is in agreement with our previous observations regarding WOX1-mediated apoptosis both *in vitro* and *in vivo* [26]–[31], [35], [38].

In addition to prostate cancer cells, we also showed C1q/WOX1-induced death of breast and neuroblastoma cells. The death event could be universal, as long as C1q and WOX1 are present in both the extracellular and intracellular milieu, and are sufficient in activating the apoptotic cascade. WOX1 is known to participate in the early event of apoptosis emanating from the membrane receptors (e.g. Fas or TNF receptor) [35].

C1q rapidly induces accumulation of activated WOX1 in the nuclei in non-transfected DU145 cells in less than 2 hr. Without serum C1q or C6, the nuclear accumulation is blocked. However, WOX1 nuclear accumulation can be restored by exogenous C1q. Thus, under physiology conditions, serum C1q and C6 sustain optimal activation of WOX1 to maintain normal cell physiology.

We have recently shown that TGF-β1 may interact with membrane hyaluronidase 2 (Hyal-2), followed by recruiting WOX1 and the resulting WOX1/Hyal-2 complex relocation to the nuclei [38]. Thus, Hyal-2 could be the potential link for C1q/WOX1 signaling. Binding of Hyal-2 with C1q remains to be determined.

C1q did not enhance p53-mediated apoptosis. C1q may suppress the synergistic effect of p53 and WOX1 in inducing apoptosis [26], [27], suggesting that a balanced p53/WOX1 binding interaction is needed to control cell survival and death in response to C1q. Most interestingly, in the absence of C9, serum causes nuclear accumulation of p53, suggesting that serum C9 controls p53 activation for regulating gene transcription *in vivo*. Upon phosphorylation at key serines such as Ser15, 20 and 46, p53 accumulates in the nuclei [30], [41], [42]. This implies that cancer cell growth and invasion can be regulated by serum complement C1q, C6 and C9 via their regulation of the activation of tumor suppressor p53 and WOX1. In the absence of serum C1q and C6, JNK1 becomes constitutively activated, suggesting that C1q and C6 block JNK1 activation *in vivo*. These above-mentioned effects do not appear to be associated with activation of the complement cascade via both classical and alternative pathways. No activated complement C3 fragments were shown to deposit on cell surface.

Human tumor suppressor gene *WWOX* encodes the mRNA for translating into WWOX/WOX1, WOX2 and other isoforms (if present) [20], [21], [43], [44]. In the absence of C1q or C6, expression of WOX2 is downregulated, whereas WOX1 expression is not affected. These observations indicate that C1q and C6 are likely to support alternatively splicing of *WWOX* mRNA, thus leading to the generation of WOX2 protein. Whether WOX2 acts as a tumor suppressor is unknown. Presumably, C1q and C6 are needed for serum-dependent cell survival by maintaining ERK activation and WOX2 expression. WOX2 may act as a dominant negative and counteracts the tumor suppressor function of wild type WWOX/WOX1 *in vivo*, thereby supporting cancer growth. Both WOX1 and WOX2 have been shown to be downregulated in the neurons of Alzheimer's disease [43]. Indeed, C1q has been implicated in the pathogenesis of neuronal death in the neurodegenerative diseases such as in Alzheimer's disease [45], [46]. There is a strong possibility that C1q activates WOX1 in neurons, which ultimately leads to cell death. Conceivably, the concentrations of C1q in the brain matrix and the cytosolic levels of neuronal WOX1 will determine the extent of C1q/WOX1 signaling and cell death.

Both WOX1 and isoform WOX2 proteins can be upregulated during the early stages of progression of breast, prostate and other types of cancers [29], [32]. Nonetheless, the C1q/WOX1 signaling could be less efficient in cancers, as many advanced cancer cells are deficient in the wild type WWOX/WOX1 [20], [21]. Serum C1q and C6 control the expression of WOX2 and may play a role in determining tumor progression. Suppressed expression of complement C6 and C7 mRNAs has been shown in patients with oesophageal carcinoma and colon and kidney cancers [47]. Functional significance of this finding relative to tumorigenesis and activation of p53, WOX1, ERK and JNK1 remains further investigation.

Complement C6 deficiency has been shown to prolong tail bleeding, along with reduced platelet aggregation, in transgenic animals [48]. Also, absence of C6 protects against sepsis mortality in rats [49]. Nonetheless, the role of serum C6 in the development of solid tumors and leukemia has never been established. In contrast, single nucleotide polymorphism in the C1qA component of complement correlates with the pattern of clinical breast cancer metastasis [50]. C1q interacts with specific membrane receptors to regulate immune responses [51], [52]. In addition, C1q interacts with a cellular membrane type-1 matrix metalloproteinase that may regulate cancer progression and metastasis [53]. Presence of membrane C6 receptor is not known and remains to be investigated.

Both HA and hyaluronidases are significantly elevated in the circulation in patients with prostate cancer [54], [55] and especially in metastatic cancers [3], [4]. While a relatively constant amount of HA is in circulation [3], [4], these HA polymers indeed regulate the physiological function of normal blood leukocytes, leukemia and cancerous cells via membrane CD44 and other receptors. Our data showed that HA tends to increase p53 relocation to the cytoplasm, suggesting that p53 can readily undergo a rapid turnover by the ubiquitination/proteasomal system in the cytoplasm. Conceivably, this mechanism to reduce wild type p53 would help facilitate prostate cancer cell growth. Interestingly, HA stimulated p53 nuclear export, when DU145 cells were cultured under serum-free or C9-free conditions. In contrast, HA induces p53 nuclear import, when cells were grown in serum without C6. Thus, serum C6 and C9 may compete for controlling the activation of p53.

Activation of STAT3 signaling pathway is critical for prostate cancer invasion [23]–[25]. Of particular interest is that HA induced phosphorylation of STAT3 in DU145 cells when cultured in the absence of C1q. These observations further imply that downregulation of C1q enhances prostate hyperplasia and cancerous formation due to failure of WOX1 activation and increased activation of STAT3.

We have shown that conformationally altered HA restricts hemolytic function of the complement classical pathway and blocks the expression of WOX1 in DU145 cells [7], [12], [13]. Serum complement proteins are likely to be restricted by HA via binding to the accessible polyanionic charges within a disrupted HA matrix rather than an intact HA matrix. Heat-induced alteration of HA conformation is likely to occur under inflammatory conditions *in vivo*. Hyaluronidase digestion definitely causes changes in HA conformation, especially in patients with metastatic cancer.

In summary, we have presented a novel functional role of complement C1q and C6 in regulating the expression of WOX2 and activation of ERK, JNK1 and WOX1. Also, complement C9 controls nuclear localization or activation of p53. HA participates in cancer progression and metastasis, and also modulates the effects of C1q and C6, along with STAT3 activation. Our findings have apparently added a new feature of complement proteins in regulating cancer cell survival and progression. We believe that the serum environment, which contains complement proteins and circulating hyaluronan, affects the behavior of cancer cells and decides the best timing for these cells to invade. Apparently, global analysis for complement protein expression in cancer cells is needed to elucidate the crucial role of complement components in regulating cancer cell progression and metastasis.

## Materials and Methods

### DU145 cells, specific complement component-depleted human sera, antibodies, and cDNA expression constructs

Human prostate cancer cell line DU145 was cultured overnight under serum-free conditions, or in the presence of 0.5 or 1% normal human serum (NHS), or ΔC1q (C1q depleted; from Sigma-Aldrich and Quidel), ΔC6 (C6 depleted; Quidel), ΔC7 (C7 depleted; Quidel), ΔC8 (C8 depleted; Quidel), or ΔC9 serum (C9 depleted; Quidel). We examined the expression and phosphorylation of ERK, WOX1, and other indicated proteins by Western blotting [26], [27]. Under stress conditions, WOX1 undergoes phosphorylation at Tyr33 both *in vitro* and *in vivo* [26], [27].

Specific antibodies against WOX1, WOX2, and its Tyr33-phosphorylated form (p-WOX1) were produced in rabbits, as described [26]–[31]. Additional specific antibodies used in this study were against the following proteins: 1) p53, JNK1, p-JNK1 (phosphorylation at Thr183 and Tyr185), p-ERK (Tyr204 phosphorylation) from Santa Cruz Biotechnologies, 2) p-STAT3 (Tyr705 phosphorylation) and STAT3 from New England BioLab, 3) ERK from BD Transduction Laboratory, and 4) C1q from Quidel. Where indicated, the cultured cells were treated for 1 hr with medical grade Healon (highly purified high-molecular-size HA; 50 µg/ml). In addition, medical grade HA used for testing was from Lifecore, a kind gift of Dr. G. Armand of the Glycomed Research, Hastings-on-Hudson, New York.

### Immunofluorescence microscopy and single cell imaging analysis

DU145 cells were cultured on cover slides under serum free conditions, or in the presence of 1% NHS, or NHS depleted with an indicated complement component, for 16–24 hr. These cells were treated with or without HA for 1 hr (50 µg/ml), followed by processing immunofluorescence microscopy using dual antibodies [26], [27]. We examined the expression and localization of ERK, WOX1, p53, STAT3 and other indicated proteins by immunofluorescence microscopy. Appropriate secondary antibodies, conjugated with Texas red or Alexa Flour, were from Rockland and Molecular Probes/Invitrogen, respectively. Nuclei were stained with DAPI (4′,6-diamidino-2-phenylindole). Single-cell imaging analysis was performed by Photoshop CS2 (Adobe) and Image-Pro Plus (Media Cybernetics). Where indicated, expression of two selected proteins in ∼200 cells in randomly specified areas was quantified. In negative controls, cells were stained with secondary antibody only. The resulting background fluorescence was used to subtract the positive signals from using a specific primary antibody staining for the cell imaging (using the Image-Pro Plus software). Data were calculated and presented as mean±standard deviation. Student's *t* test was used for statistical analyses.

### Human postmortem prostate tissues

We obtained medical and legal consent to acquire postmortem prostate tissues from the autopsy service at the Department of Pathology, University of Colorado Health Sciences Center (by CI Sze). The tissues included patients with benign prostatic hypertrophy (BPH), prostatic adenocarcinoma, and age-matched normal prostate tissues (5 each). De-parafinization and immunofluorescence staining were performed similarly as described above [29], [43]. Primary antibodies used in the immunofluorescence were against: C1q (Quidel) and p-WOX1 [27]. Secondary antibodies were anti-rabbit IgG FITC and anti-mouse IgG Texas Red. In negative controls, secondary antibodies were used for staining only.

### Cell cycle analysis, BCC stable transfectants, and time-lapse microscopy of live cells

DU145 cells were transfected with a WOX1-p-EGFPC1 or an “empty” p-EGFPC1 construct by electroporation (20 µg of DNA/3×10^6^ cells; 200 Volt and 50 millisecond; BTX ECM 830 Square Wave Electroporator, Genetronics) [26], [27], followed by culturing for 16–24 hr. These cells, with transient expression of fluorescent proteins, were then treated with purified C1q (1–5 µg/ml; from Quidel and Sigma-Aldrich with similar purity and potency) or medium alone for 8–24 hr. Propidium iodide staining and cell cycle analysis were carried out using a fluorescence-activated cell sorting (FACS)/flow cytometery machine (BD) to measure the extent of apoptosis or growth inhibition [27]. The extent of green fluorescence was normally greater than 60%. Cell death caused by electric shock from electroporation was gated out, and the rest of the cell populations were subjected to analyses. A dominant negative WOX1 construct was developed [27], and used to block C1q-mediated cell death. Where indicated, DU145 cells-expressing EGFP-WOX1, EGFP-WOX1ww (WW domains only), or EGFP were treated with C1q (1 µg/ml) and subjected to time-lapse microscopy at 37°C and 5% CO2/atmosphere conditions for determining cell morphological changes, including cell shrinkage, membrane blebbing, and nuclei condensation, using a Nikon Eclipse TE-2000S inverted microscope. In addition, we established stable transfectants of skin basal cell carcinoma (BCC) [56] for expressing EGFP or EGFP-hWOX1 (human WOX1/WWOX). These cells were established from G418 selection [26], [27]. Time-lapse microscopy was performed using these cells to determine C1q-mediated cell death.

### Total internal reflection fluorescence (TIRF) microscopy

TIRF microscopy utilizes evanescent wave to selectively measure the cell surface and cytoskeletal areas of approximately 100 nm in depth. Surface plasmon-enhanced two-photon fluorescence microscopy for live cell membrane imaging was developed, as described [37], [57]. A Nikon Eclipse microscope was modified for measuring fluorescent signals on the cell surface [37], [57]. DU145 cells were cultured on pieces of cover glass overnight, followed by exposure to C1q (1 µg/ml) for 1 hr. The live cells were imaged, or they were fixed with 4% paraformaldehyde for imaging.

## Supporting Information

Figure S1 Complement C1q increases WOX1-induced apoptosis of DU145 cells (a dose-related experiment). (A) DU145 cells were transfected with various amounts of EGFP-WOX1 (tagged with EGFP; 2.5–10 µg) by electroporation. The cells were then cultured overnight and treated with purified C1q (1 µg/ml) for 24 hr. (B,C) C1q enhanced WOX1-induced apoptosis of DU145 cells (see the increases in SubG1 phase but decreases in the G0/G1 phase). A representative data set (bar graphs) is shown from 3 experiments. In control experiments, C1q did not enhance apoptosis in DU145-overexpressing EGFP (data not shown). In positive controls, non-transfected DU145 cells were treated with staurosporine (1 µM) for 8 hr, prior to cell cycle analysis. WOX1: EGFP-WOX1.
(0.40 MB TIF)

Figure S2 Complement C1q enhances ectopic WOX1-induced apoptosis of breast MCF7 cells. (A) MCF7 cells were transfected with EGFP-WOX1 or EGFP alone by electroporation. Following overnight culture, these cells were treated with purified C1q (1 µg/ml) for 24 hr. C1q enhanced apoptosis of MCF7 cells transiently overexpressing EGFP-WOX1. In controls, C1q did not enhance apoptosis in MCF7-overexpressing EGFP (data not shown). In positive controls, non-transfected MCF7 cells were treated with staurosporine (1 µm) for 8 hr, followed by cell cycle analysis. In another control, the cells received medium only. The number on the top of each bar is the percentage of cell numbers (bar graphs at right). (B,C) Shown in the bar graphs is a representative data set from 3 experiments. WOX1: EGFP-WOX1. Control: cells without electroporation. Stauro: cells treated with staurosporine.
(0.40 MB TIF)

Figure S3 Complement C1q enhances ectopic WOX1-induced apoptosis of neuroblastoma SH-SY5Y cells. (A) SH-SY5Y cells were transfected with EGFP-WOX1 or EGFP alone by electroporation. Following overnight culture, these cells were treated with purified C1q (1 µg/ml) for 24 hr. C1q enhanced apoptosis of SH-SY5Y cells overexpressing EGFP-WOX1. In controls, C1q did not enhance apoptosis in SH-SY5Y-overexpressing EGFP (data not shown). (B,C) A representative data set is shown as bar graphs from 3 experiments. WOX1: EGFP-WOX1.
(0.68 MB TIF)

Figure S4 Complement C1q enhances ectopic WOX1-induced internucleosomal DNA fragmentation of DU145 and SH-SY5Y cells. (A) DU145 cells were electroporated with the indicated vectors and then cultured for 24 hr, followed by exposure to purified human C1q (1 µg/ml) for 8 hr. C1q increased DNA fragmentation in WOX1-expressing DU145 cells. In negative controls, cells were electroporated with nothing, or witout electroporation (no ep). Staurosporine (1 µM)-treated cells were regarded as positive controls. (B) Similar results were obtained by testing SH-SY5Y cells.
(1.52 MB TIF)
