# Supplementary figures and images for: Complement C1q Activates Tumor Suppressor WWOX to Induce Apoptosis in Prostate Cancer Cells
Source: PLoS One. 2009 Jun 1;4(6):e5755. doi: 10.1371/journal.pone.0005755 (PMC2685983; doi:10.1371/journal.pone.0005755)

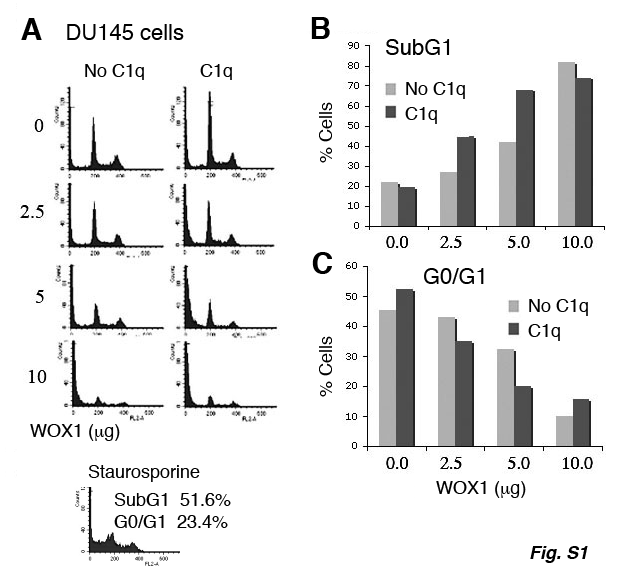

Supplement: Figure S1 — Complement C1q increases WOX1-induced apoptosis of DU145 cells (a dose-related experiment). (A) DU145 cells were transfected with various amounts of EGFP-WOX1 (tagged with EGFP; 2.5–10 µg) by electroporation. The cells were then cultured overnight and treated with purified C1q (1 µg/ml) for 24 hr. (B,C) C1q enhanced WOX1-induced apoptosis of DU145 cells (see the increases in SubG1 phase but decreases in the G0/G1 phase). A representative data set (bar graphs) is shown from 3 experiments. In control experiments, C1q did not enhance apoptosis in DU145-overexpressing EGFP (data not shown). In positive controls, non-transfected DU145 cells were treated with staurosporine (1 µM) for 8 hr, prior to cell cycle analysis. WOX1: EGFP-WOX1. (0.40 MB TIF) [file pone.0005755.s001.tif]

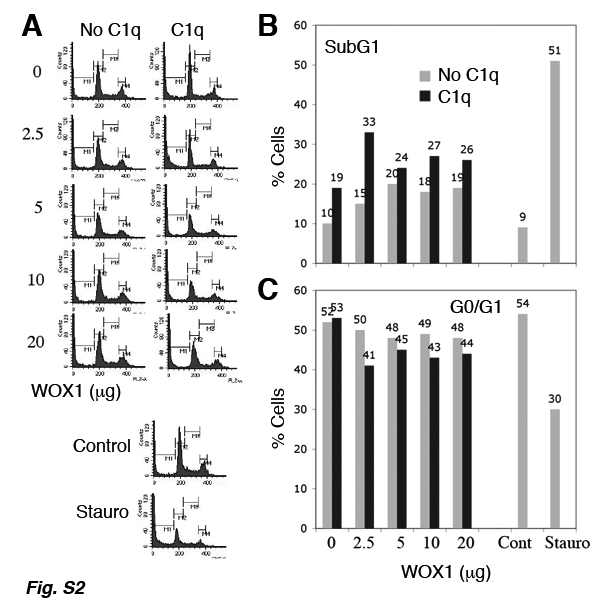

Supplement: Figure S2 — Complement C1q enhances ectopic WOX1-induced apoptosis of breast MCF7 cells. (A) MCF7 cells were transfected with EGFP-WOX1 or EGFP alone by electroporation. Following overnight culture, these cells were treated with purified C1q (1 µg/ml) for 24 hr. C1q enhanced apoptosis of MCF7 cells transiently overexpressing EGFP-WOX1. In controls, C1q did not enhance apoptosis in MCF7-overexpressing EGFP (data not shown). In positive controls, non-transfected MCF7 cells were treated with staurosporine (1 µm) for 8 hr, followed by cell cycle analysis. In another control, the cells received medium only. The number on the top of each bar is the percentage of cell numbers (bar graphs at right). (B,C) Shown in the bar graphs is a representative data set from 3 experiments. WOX1: EGFP-WOX1. Control: cells without electroporation. Stauro: cells treated with staurosporine. (0.40 MB TIF) [file pone.0005755.s002.tif]

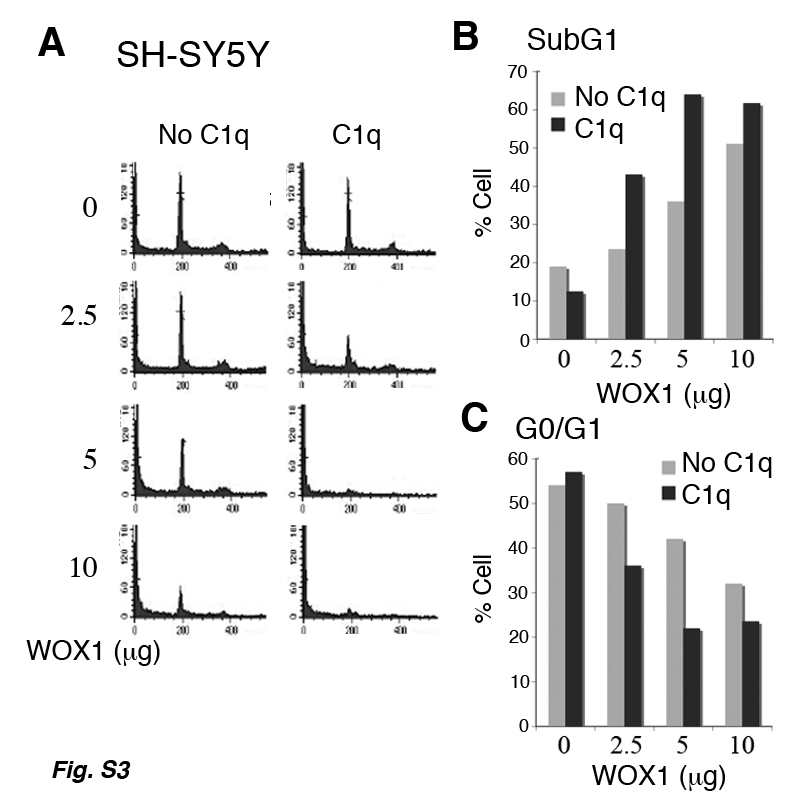

Supplement: Figure S3 — Complement C1q enhances ectopic WOX1-induced apoptosis of neuroblastoma SH-SY5Y cells. (A) SH-SY5Y cells were transfected with EGFP-WOX1 or EGFP alone by electroporation. Following overnight culture, these cells were treated with purified C1q (1 µg/ml) for 24 hr. C1q enhanced apoptosis of SH-SY5Y cells overexpressing EGFP-WOX1. In controls, C1q did not enhance apoptosis in SH-SY5Y-overexpressing EGFP (data not shown). (B,C) A representative data set is shown as bar graphs from 3 experiments. WOX1: EGFP-WOX1. (0.68 MB TIF) [file pone.0005755.s003.tif]

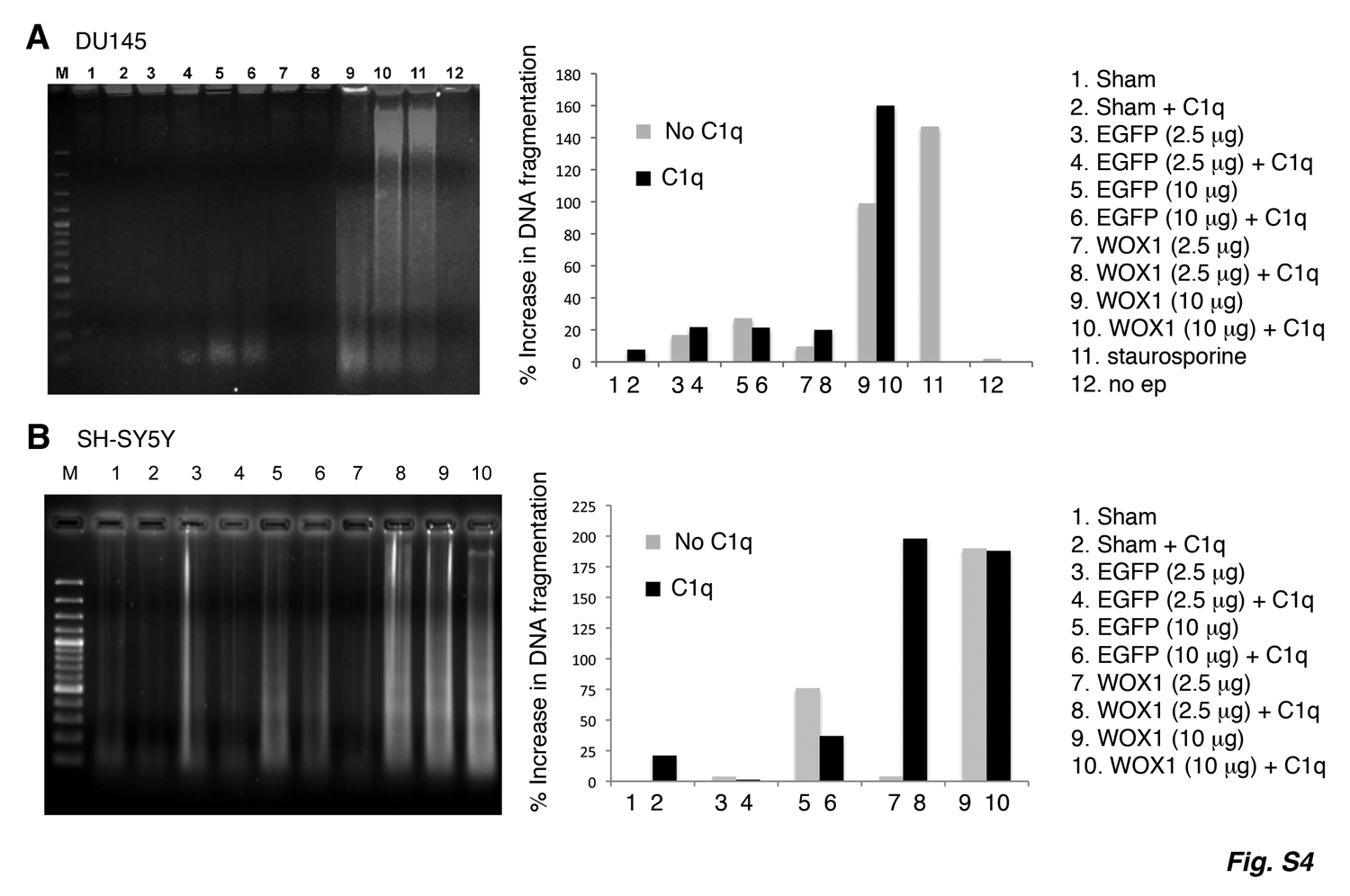

Supplement: Figure S4 — Complement C1q enhances ectopic WOX1-induced internucleosomal DNA fragmentation of DU145 and SH-SY5Y cells. (A) DU145 cells were electroporated with the indicated vectors and then cultured for 24 hr, followed by exposure to purified human C1q (1 µg/ml) for 8 hr. C1q increased DNA fragmentation in WOX1-expressing DU145 cells. In negative controls, cells were electroporated with nothing, or witout electroporation (no ep). Staurosporine (1 µM)-treated cells were regarded as positive controls. (B) Similar results were obtained by testing SH-SY5Y cells. (1.52 MB TIF) [file pone.0005755.s004.tif]
